# Review of “Introduction to BioMEMS” by Albert Folch

**DOI:** 10.1186/1475-925X-12-25

**Published:** 2013-03-25

**Authors:** Joel A Kubby

**Affiliations:** 1 Department of Electrical Engineering, University of California Santa Cruz, California, USA

## Abstract

This article is a review of the book “Introduction to BioMEMS” by Albert Folch which is published by CRC Press, Taylor & Francis Group. It will review the contents of the book and discuss it suitability as textbook, highlights of the book, and comparison to other textbooks on BioMEMS.

## Book details

2013: List Price. $89.95, ISBN number: 978-1-4398-1839-8

## Summary

“Introduction to BioMEMS” by Albert Folch is a well written textbook for advanced undergraduate and introductory graduate level courses in biological applications of micro-electro-mechanical systems and lab-on-a-chip devices. It provides coverage of both historical perspectives and the latest developments in this rapidly developing field. The extensive use of color figures, the non-extensive use of equations, and a thorough glossary of terms will make this textbook ideal for an interdisciplinary introductory bioengineering course. The on-line price of the book ranges from $84-$90.

## Suitability as a course textbook

The extensive use of high-quality color figures and the minimal use of equations will be helpful to interdisciplinary bioengineering students who will most likely use this as a textbook in an introductory course. Folch points out that he has not written a comprehensive textbook that is full of references to the literature, but rather an introductory textbook that is designed to guide students through the myriad of BioMEMS devices, focusing attention on the highest impact and most creative work in the field. The book has over a decade of use in the authors own courses at the University of Washington’s Bioengineering Department, so it has been well tested in the classroom and ready for use by new instructors in their own courses. The provision of an extensive glossary of terms that are highlighted when used in the text will help new students overcome the challenges of specialized jargon that is typical of engineering fields.

## Framework and contents of the book

The book begins with an overview of microfabrication techniques for both hard and soft materials followed by an extensive review of microfluidics. In these introductory chapters the author details the benefits of microfabrication (smaller, faster, and cheaper). The chapter on microfluidics is very extensive covering many different designs of fluidic devices (actuators, valves, pumps, mixers and plumbing) and includes practical details such as how to fill microfluidic channels and visualize fluid flow. Following these introductory materials are the different platforms that can be fabricated using this toolbox, including molecular biology (sample preparation, assays, genomics and proteomics, DNA processing, analysis) and cell based lab-on-a-chip applications (cell sorting, trapping, culture, bioreactors), cellular biology (cell-substrate and cell-cell communications, migration, neurobiology, developmental biology, yeast and plant cell biology, cell dynamics), tissue microengineering (scaffolds, co-cultures, stem cells, morphogenesis) and implantable microdevices (electrodes, needles and tools for surgery). While the book is designed for a one semester course, the book can be used for a one quarter course by first covering the introductory materials and then selecting some of the later detailed chapters for a more focused course.

## Comparison to other BioMEMS textbooks

I have considered a number of different textbooks for use in my graduate level class on BioMEMS at UC Santa Cruz, including “Biomedical Microsystems” by Ellis Meng [1], “Bio-MEMS Technologies and Applications” edited by Wanjun Wang and Steven A. Soper [2], both of which are published by CRC Press, Taylor and Francis Group. “Biomedical Microsystems” by Ellis Meng [1] covers much of the same introductory subjects in materials, microfabrication methods and microfluidics, but the later chapters differ somewhat in the applications of these topics. “Introduction to BioMEMS” by Albert Folch has more of a bioengineering focus in the later chapters, covering subjects such as molecular biology on a chip and cell-based chips, BioMEMS for cell biology and tissue engineering. “Biomedical Microsystems” by Ellis Meng [1] has more of a device focus, covering focused topics like sensing and detection methods in a dedicated chapter. “Bio-MEMS Technologies and Applications” is a compilation of chapters contributed by different authors, which is more suitable for an advanced course where the flow of subject matter is not as smooth as an introductory text written by a single author, but provides detailed information that is useful to advanced students and practitioners who have already been introduced to the field. One clear difference between these three books is the excellent and current overview of the field of microfluidics that is provided by Folch. The well-illustrated color chapters bring the subject matter to life. The minimal use of equations throughout “Introduction to BioMEMS” by Albert Folch will also be helpful to bring interdisciplinary bioengineering students onto a level playing field.

## Highlights

The book has an incredible collection of color figures, many of which are the original figures from seminal papers in the field. If a picture is worth a thousand words, this text has over 400,000 words in the figures alone! The author has made available his slides that are based on these figures which will be very helpful to instructors adapting this book for their courses. Providing the original Power Point slides, rather than PDF copies of the slides, will enable instructors to customize the slides for their particular curriculum. The glossy paper that is used throughout the text is higher quality and provides higher print contrast for easier reading than the paper that is used in normal text books, where only the color plate sections uses glossy paper.

Another feature of the book is the detailed appendices that include teaching materials such as suggested exercises for each of the eight chapters, tips for test taking in BioMEMS courses based on the instructors past experiences teaching the course, microfluidics outreach and education for K-12 students, a short pictorial overview of the BioMEMS history, and a thorough glossary of technical terms that appear in the text. The pictorial overview will be a useful broad introduction to the field of BioMEMS to those who are interested in learning about the field, but do not have time to take the course. It will also be a good “hook” to catch the interest of students in the introductory lecture, and as a final review of the many subject areas that are covered in more detail throughout the chapters. The glossary of terms will be very helpful to newcomers to the field, to overcome the specialized jargon that can be a barrier to entry for many.

## Minor criticisms

While the plethora of color figures is a definite feature of this book, they also give the book somewhat of an odd odor, sort of like a paint store.

I have mixed feelings about the exercises that are included. Some of them merely ask the students to hunt down and regurgitate information in the chapter. I have found that students do not really appreciate these sorts of questions and they are somewhat boring to grade, as each students answers are all the same. Nonetheless some of the questions are very good, particularly the ones that have been labeled as “design challenges”. These exercises will encourage independent thinking on the part of the students and I have found that they really enjoy these open ended questions that allow for creative answers instead of regurgitation. I have found these types of questions are very interesting to grade and provide useful feedback to the instructor on how well the students are learning useful information. They also provide an opportunity for instructors to learn from their students (when one teaches, two learn).

## Conclusions

I highly recommend this book for an advanced undergraduate or entry level interdisciplinary graduate course in BioMEMS. It is well written and produced. The color figures differentiate it from other textbooks in this area and the author has done a thorough job documenting the history and current practices in this rapidly developing field. I plan to use it in my own class.

## Abbreviations

DNA: Deoxyribonucleic acid; MEMS: Micro-Electro-mechanical systems; PDF: Portable document format.

## Competing interests

The author has written a guide book on Hands-On MEMS design, but it does not compete with this textbook on BioMEMS.
